# The Influence of Myofascial Techniques on the Range of Motion and Flat Foot Efficiency in Adults with Symptomatic Flat Foot: A Controlled Randomised Trial

**DOI:** 10.3390/healthcare13162046

**Published:** 2025-08-19

**Authors:** Sabina Kaczor, Urszula Żmudzińska, Aleksandra Kulis

**Affiliations:** Faculty of Motor Rehabilitation, The University of Physical Culture in Krakow, al. Jana Pawła II 78, 31-571 Krakow, Poland; sabkaczor@gmail.com (S.K.); urszula.zmudzinska@awf.krakow.pl (U.Ż.)

**Keywords:** flat foot, myofascial release, range of motion, functional efficiency, adults

## Abstract

**Objective**: Symptomatic flat foot is quite a common pathology in adults. Myofascial release is one of the physiotherapeutic methods that are currently very often used in the treatment of musculoskeletal diseases. This study aimed to assess the impact of myofascial release on the range of motion and functional efficiency of the flat foot in adults. **Method**: The study involved 60 people with flat feet allocated to four groups and subjected to therapy lasting four weeks: group MRE (Myofascial Release and Exercises; 15 people): myofascial techniques and an exercise programme; group MR (Myofascial Release; 15 people): only myofascial techniques; group E (Exercises; 15 people): only an exercise programme; and the control group C (Control; 15 people): no intervention. Goniometric measurements of the range of motion of the ankle joint and the Foot and Ankle Outcomes Questionnaire (FAOQ) were used to evaluate the effects of the therapy. **Results**: The range of all tested movements significantly improved after therapy in both feet simultaneously in groups MRE (left foot: dorsiflexion *p* = 0.017; plantar flexion *p* = 0.006; inversion *p* = 0.003; and eversion *p* = 0.001; right foot: dorsiflexion *p* = 0.008; plantar flexion *p* = 0.003; inversion *p* = 0.008; and eversion *p* = 0.004) and MR (left foot: dorsiflexion *p* = 0.001; plantar flexion *p* = 0.001; inversion *p* = 0.001; and eversion *p* = 0.001; right foot: dorsiflexion *p* = 0.001; plantar flexion *p* = 0.002; inversion *p* = 0.001; and eversion *p* = 0.029). The FAOQ results were significantly better after therapy in groups MRE (*p* = 0.010), MR (*p* = 0.001) and E (*p* = 0.015). **Conclusions**: In the people studied, the combination of myofascial techniques and exercises (MRE) was the most effective for improving the tested ranges of motion of the ankle joint. Myofascial techniques had a significant impact on the performance of the feet assessed with the FAOQ.

## 1. Introduction

Adult flatfoot is a common disorder that typically affects middle-aged and elderly women [1]. In the United States five million patients are currently diagnosed with flatfoot, and in the UK the prevalence is estimated to be over 3% in women over 40 years old. Furthermore, 10% of the geriatric population suffer from severe acquired flatfoot due to the degeneration of muscle mass and bone structure [2]. The problems, aetiology, diagnosis and treatment of flat feet are often discussed by researchers, but a significant number of their publications refer to the population of children and adolescents [3,4,5]. Meanwhile, it should not be forgotten that this dysfunction also applies to adults who, in addition to having a flattened longitudinal arch, may also develop other disorders, such as a limited range of motion of the ankle joint and foot joints, as well as defects or degenerative changes in the tendons and ligaments in some foot and shin muscles [6]. Lowering the longitudinal arch of the foot may also affect the biomechanics of the lower limb [7,8] which results in an excessive overload of bone elements and soft tissues [9]. Not only pain but also a limited range of motion and weakened muscle strength occur. These structural changes and symptoms adversely affect functional efficiency, which in turn lead to the creation of compensatory mechanisms that, in a vicious circle, deepen the symptoms and dysfunctions in the foot [10,11,12].

The standard nonsurgical treatments for flat feet are exercises and orthotics [2,13], while surgical treatment includes numerous combinations of procedures, including osteotomies of selected bones, arthrodeses of selected joints, ligament repairs, and Achilles tendon lengthening [6]. However, flat feet in adults is a problem that is not easy to treat, which is why physiotherapists constantly look for new, effective techniques that can reduce or eliminate the symptoms associated with this dysfunction. Myofascial release is an increasingly popular method of working with a patient and includes many techniques focusing on soft tissues. Myofascial release is widely used in the treatment of musculoskeletal dysfunctions, particularly of pain and a limited range of motion of joints [14]. In some studies, the use of myofascial release in dysfunctions of the distal part of the lower limb proved to have a beneficial effect on the range of motion of the ankle joint, among others, but these works concern mainly young and physically active people [15,16]. Most authors examining the effectiveness of myofascial release in the distal part of the lower limb agree that this method has great potential [9,17]. However, among these reports, there are no studies assessing the effectiveness of myofascial release in the treatment of flat foot dysfunctions, which merits research. Therefore, this study aimed to assess the impact of myofascial release on the range of motion and flat foot efficiency in adults. We hypothesized that myofascial release may improve range of motion and flat foot efficiency in adults.

## 2. Material and Methods

### 2.1. Participants

Sixty randomly selected people a flattened longitudinal arch of the foot and reported foot pain qualified to participate in the study (see Table 1). The study included 47 women (78.3%) and 13 men (21.7%). They were randomly (computer draw; block randomization; main author) divided into four groups:

- group MRE (15 people), subjected to a four-week rehabilitation programme including myofascial techniques and a set of exercises performed every day;
- group MR (15 people) was subjected to a four-week rehabilitation programme including only myofascial techniques;
- group E (15 people) was subjected to a four-week rehabilitation programme based on exercises performed every day throughout the project;
- group C (15 people) was a control group (no intervention).

In each group, the number of women was higher. The gender distribution of respondents did not differ statistically significantly in the four groups (*p* = 0.329). Patients were not informed about which treatment they were receiving after being assigned to a specific group. Likewise, the people taking measurements of the subjects did not know to which group each patient belonged.

**Figure 1:**
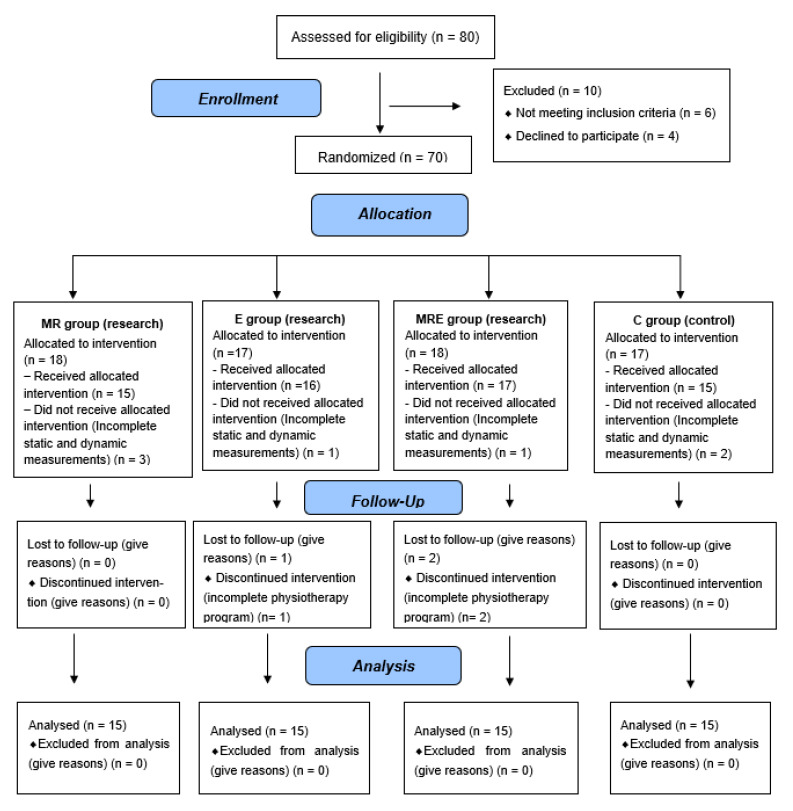
Consort diagram.

Inclusion criteria (Figure 1):

− flat foot (unfixed changes)
− aged 20 to 49 years
− pain in the foot
− no injuries in the last six months affecting the functioning of the lower limbs
− no surgeries in the last eighteen months on the lower limbs
− no previous Achilles tendons pathology
− no previous Achilles tendon medication
− no neurological, metabolic, rheumatic or orthopaedic diseases
− no contraindications to therapy
− written consent to participate in the study

The study was approved by the Bioethics Committee at the Regional Medical Chamber in Krakow (No. 94/KBL/OIL/2016, approval date: 14 June 2016).

Measurements were carried out at the University of Physical Education in the Faculty of Motor Rehabilitation, in collaboration with Medical Centre Liszki. The study was conducted in accordance with the Code of Ethics of the World Medical Association (Declaration of Helsinki 1964). Informed consent was obtained for each patient.

This study was registered in the Australian and New Zealand Clinical Trials Registry. Registration number: ACTRN12617000257369 (registration date: 20 February 2017). Patients’ written consent was obtained, and the rights of subjects were protected.

### 2.2. Assessment Tools

To determine the basic morphological indicators, body weight and body height were measured once. All patients (regardless of group membership) had their ankle joint range of motion measured twice using a goniometer and functional efficiency using the Foot and Ankle Outcomes Questionnaire (FAOQ). In groups MRF, MF and E, the first measurement took place before the start of the physiotherapeutic programme and the second after its completion (four weeks later). In control group C, two measurements were also made and the time interval between them was four weeks.

Body height measurement—body height was measured using a Martin-type anthropometer (Seritex, New York, NY, USA) with an accuracy of 0.1 cm. A patient’s height was measured from the top of the head (vertex) in the horizontal plane to the plantar plane of the feet (basis) [18].

Body weight measurement—body weight was determined using a Tanita scale (Tanita Corporation, Tokyo, Japan) with an accuracy of 0.1 kg [19].

Body fatness measurement—this measurement was performed using the BMI, the most popular indicator in the world for assessing body fatness, calculated according to the formula weight/height^2^ (kg/m^2^) [20].

Goniometric examination—the range of motion of the ankle joint of each study participant was measured twice using a goniometer. To measure dorsiflexion and plantar flexion, each patient sat on a couch with the lower legs hanging freely. The goniometer axis was placed on the lateral ankle, the stationary arm was positioned along the fibula (aiming at the fibula head), and the moving arm was positioned along the fifth metatarsal bone. The norms for dorsiflexion and plantar flexion of the ankle joint were 15° to 20° and 45° to 50°, respectively. To measure the inversion movement, the axis of rotation was the head of the fifth metatarsal bone. The stationary arm was positioned parallel to the lateral edge of the lower leg, and the moving arm was positioned along the heads of the metatarsal bones. Moreover, 40° was assumed as the norm for the inversion movement. For the eversion movement, the rotation axis was located on the head of the first metatarsal bone. The moving arm was positioned as before along the heads of the metatarsal bones, while the stationary arm was positioned along the medial edge of the lower leg. The assumed norm for the eversion movement was 20° [21].

FAOQ—all participants were assessed twice with the FAOQ (Polish version), which is a tool used to assess and track treatment results in the ankle and foot area based on the patient’s subjective assessment. The questionnaire consists of 25 questions organized into five subscales: pain assessment (9 questions), function assessment (6 questions), stiffness and swelling assessment (2 questions), stability assessment (3 questions), and shoe comfort assessment (5 questions). The assessment covers the last 7 days. Respondents select one response on a scale of 1 to 5 or 1 to 6. Lower values indicate better functional status, higher values indicate worse. Results, standardized to percentages, are reported on a scale of 0 to 100 [22].

### 2.3. Intervention

Myofascial release—groups MRE and MR participated in myofascial therapy sessions. The meetings took place for four weeks, twice a week, and lasted 40 min (20 min for each lower limb). The purpose of this technique was to reduce pain and increase muscle mobility and flexibility. The so-called direct techniques of myofascial therapy included the following:

- Lengthening of the peroneal muscles—the patient is lying on his side (untreated). The therapist uses his fingers to move along the peroneal muscles in the direction of their elongation, working from the lateral ankle towards the head of the fibula.
- Lifting of the plantar flexors (the gastrocnemius, and the soleus muscle)—the patient is facing forward. The therapist, using two hands, covers the muscles and gently lifts them. Then it introduces rotations along the long axis of the lower leg. The therapist keeps rotating, waiting for relaxation, then returns to starting position. The therapist repeats the same technique with a bent knee joint.
- Working to elongate the gastrocnemius and soleus calf muscles—the patient is facing forward, the feet are outside the couch. The therapist moves an open hand or fist over the muscles, from the heel to the knee fossa. With the other hand or thigh, it increases the range of dorsiflexion of the ankle. The therapist repeats the same technique with a bent knee joint.
- Working on the Achilles tendon—the patient is facing forward, the feet are outside the couch. The therapist uses the knuckles to work on both sides of the Achilles tendon, introducing lengthening movements. During this work, the therapist deepens the dorsiflexion of the ankle joint.
- Working on the tissues around the heel—the patient is facing forward, the feet are outside the couch. The therapist works on both sides of the ankle joint. Using their fingers or knuckles, they slowly move the tissue from the ankles down to the heel lining. The therapist mobilizes the tissues around the heel by placing their hands perpendicular to the long axis of the shin.
- Working on plantar fascia—the patient is lying on his stomach. The therapist uses their fist and knuckles to make slow movements from the heel towards the toes, focusing on the mobilization of the tissues under the sole.
- Working on the furrows (the peroneal muscle/the soleus muscle, the gastrocnemius/the soleus muscle, the Achilles tendon/tendon crossing)—the therapist tries to separate the above-mentioned structures with the tips of a few fingers or a thumb.

Exercises—groups MRE and E performed a set of exercises every day for four weeks (from Monday to Friday) under the supervision of a physiotherapist. The exercise set included seven exercises, divided into two parts. One part consisted of exercises stretching selected muscles of the lower leg:

- Elongation of the gastrocnemius muscle:
  Starting position: standing with both feet, feet parallel to hip-width.
  Exercise: lunge forward, hands resting on the wall/table top; shifting the weight of the body forward (the heel of the lagging leg is in contact with the ground all the time) until the feeling of stretching. Maintaining the position for 20 s.
- Elongation of the soleus calf muscle:
  Starting position: standing with both feet, feet parallel to the hip-width.
  Exercise: lunge forward, hands resting on the wall/table top; bending the knee of the back leg, shifting the body weight forwards (the heel of the lagging leg is in contact with the ground all the time), until the feeling of stretching. Maintaining the position for 20 s.
- Rolling of the plantar fascia:
  Starting position: standing with both feet, feet parallel to the hip-width, under one foot at the height of the metatarsal bones, a roller or a tennis ball.
  Exercise: slowly rolling the foot with pressure, over the roller (tennis ball) towards the heel.
- Stretching of the plantar fascia:
  Starting position: sit on a chair, leg crossed; one hand grips the heel, the other hand grips the foot at the height of the metatarsal heads.
  Exercise: performing dorsiflexion of the fingers. Holding position for 20 s.
- Stretching of the peroneal muscles:
  Starting position: sit on a chair, leg crossed; one hand grips the heel, the other hand grips the foot at the height of the metatarsal heads.
  Exercise: performing foot supination. Maintaining the position for 20 s.
- Short foot exercises:
  Starting position: sit on a chair, foot (barefoot) resting on the ground (hip joint, knee joint and foot in one line).
  Exercise: simultaneous pulling of the head of the 1st and 5th metatarsal bones towards the heel. Maintaining the position for 10 s.
- Strengthening of the flexors longus:
  Starting position: standing with both feet, feet parallel to the hip-width.
  Exercise: leaning the body forward without lifting the heels and bending the waist until the patient feels a load on the front of the feet. Maintaining the position for 10 s.

The second part included exercises strengthening the tibialis posterior muscle, toe flexor muscles and short intrinsic muscles of the plantar side of the foot. The duration of therapy was 30 min a day.

**Statistical methods**—Statistical analysis of the gathered data was performed using Statistica 10.0 (StatSoft, Tulsa, OK, USA). The following parameters were used: mean average, median, minimal and maximal values and standard deviation. Normal values were verified with the Shapiro–Wilk test. For statistical analysis, the Student’s *t*-test, ANOVA test, Wilcoxon signed-rank test and Mann–Whitney U test were used. In all the tests, the level of significance was set as *p* < 0.05. In all the tests, the level of significance was set as *p* < 0.05. The Cohen’s d coefficient value was interpreted as *d* = 0.2—small effect (the observed difference is small and may have limited clinical significance), *d* = 0.5—medium effect (the observed difference is noticeable and may have moderate clinical significance), *d* = 0.8—large effect (the observed difference is significant and probably has a significant impact on clinical practice).

## 3. Results

The first correlation analysed was between the range of motion of dorsiflexion and plantar flexion of the left and right feet and the physiotherapeutic methods applied. In the left foot, the range of dorsiflexion motion improved significantly after therapy in groups MRE, MR and E, and a significant relationship in the range of plantar flexion occurred only in groups MRE and MR. In intergroup comparisons of left foot dorsiflexion after therapy, a significantly greater correlation was obtained between the results of group MRE compared to MR group and of group MR compared to group C. As regards plantar flexion, a significantly greater correlation was found between the results of group MRE compared to all other groups, and of group MR compared to group E.

In the right foot, the range of dorsiflexion motion improved significantly after therapy in groups MRE and MR, and there was a significant correlation for plantar flexion in groups MRE, MR and E. In intergroup comparisons of dorsiflexion of the right foot after therapy, a significantly greater correlation was obtained between the results of groups MRE and MR and of group MR and groups E and C, while of plantar flexion between the results of group MRE and all other groups (see Table 2).

Another analysed correlation was between the range of inversion and eversion of the left and right foot and the physiotherapeutic methods used. In the left foot, the range of motion, both inversion and eversion, improved significantly after therapy in groups MRE and MR. In intergroup comparisons of inversion of the left foot after therapy, no correlation was obtained between the results of all groups, while of eversion, a statistically significant relationship was observed between groups MRE and MR and group C.

In the right foot, both inversion and eversion improved significantly after therapy in groups MRE, MR and E. In intergroup comparisons of inversion after therapy, no correlation was obtained between the results of all groups; however, as regards eversion, a statistically significant relationship was observed only between group MRE and group MR (see Table 3).

The results of the assessment of general foot performance using the FAOQ showed a statistically significant correlation between the measurements in groups MRE, MR and E. Although improvements were observed in all groups, intergroup differences were not confirmed (see Table 4).

## 4. Discussion

Myofascial release is a method of manual work aimed at eliminating restrictions in the fascia and restoring its proper properties. In recent years, myofascial release has become one of the most frequently used methods for treating dysfunctions of the musculoskeletal system, including the foot and ankle joint, and particularly those related to plantar heel pain [23,24,25]. However, the available literature still lacks reports confirming the effectiveness of these techniques in the treatment of problems related to flat feet in adults.

In our study, we assessed the impact of myofascial release on the range of motion and functional ability of flat feet in adults. The most effective therapy for improving ankle range of motion was a combination of myofascial release techniques and exercises. Myofascial release techniques also had a significant impact on foot function, as assessed using the FAOQ questionnaire, in all study groups.

One of the problems that arise with flat feet in adults, in addition to the flattening of the longitudinal arch, is a limited range of motion within the ankle joint [6]. The literature comments widely on the influence of myofascial release on the range of foot dorsiflexion, but not in relation to coexisting flat feet. Stanek et al. [17] examined 44 physically active people whose range of motion in the ankle joint was limited to below 30°. They used a single session of compressive myofascial release and compared its effects with a single session of the Graston technique (myofascial release using a stainless-steel device instead of a therapist’s hand). They measured the range of motion of foot dorsiflexion using an inclinometer and showed that myofascial release increased the tested range of motion more effectively than the Graston technique. Škarabot et al. [26] examined 11 teenage athletes who used static stretching, foam rolling (as a form of self-myofascial release) and both methods simultaneously. The studied persons exercised 30 min a week for six months. The results showed that the passive range of motion of foot dorsiflexion was most effectively improved by both techniques used simultaneously. Garcia-Gutierrez et al. [27] applied a single session of foam rolling with vibration, which consisted of three repetitions of 20 s each. The therapy involved 38 students. The authors have shown that the use of vibrations together with foam rolling improves the range of foot dorsiflexion. Godwin et al. [28] have presented similar results. They examined 25 young people who underwent two treatments performed one week apart. The treatments included self-myofascial release (foam rolling) combined with a dynamic warm-up (five-minute ride on a stationary bike), which the authors compared to dynamic warm-up alone. As in the case of previous studies by other authors, the range of foot dorsiflexion also increased here. Lyu et al. [16] also examined young and physically active people (20 students) who were subjected to self-myofascial release (foam rolling) with vibration and dynamic muscle contractions (heel up and down exercises). The therapeutic intervention included two sessions during which patients performed two times ten repetitions of dynamic exercises and four minutes of rolling with vibration. The authors have shown that this therapy effectively increases the range of dorsiflexion and plantar flexion of the foot. Also, according to Guillot et al. [15], self-myofascial release in the form of foam rolling improves the range of foot dorsiflexion. The researchers subjected 30 professional rugby players to a therapeutic intervention, consisting of 15 sessions over seven weeks. During a single session, the tested players performed rolling for 20 s (group 1) and 40 s (group 2) or rode a stationary bike (control group). The results have shown that self-myofascial release is effective in increasing the tested range of motion regardless of the duration of the intervention. Akter et al. [23] compared the impact of myofascial release to Structural Diagnosis and Management in the treatment of plantar heel pain. The study involved 64 people aged 30 to 60 who underwent, in addition to the methods described, stretching exercises, ice compression and ultrasound therapy. The study lasted for four weeks (12 sessions), and the results showed that both methods effectively increased the range of motion of dorsiflexion and plantar flexion of the foot. An interesting study was conducted by Gupta et al. [29] who assessed the effectiveness of four weeks of myofascial release performed using the NordBlade 2.0 device compared to static stretching in 60 young men with a valgus foot. The study participants were assigned to two experimental groups and one control group. The first experimental group underwent myofascial release for 5–10 min, preceded by cryotherapy and completed with stretching exercises. The second group underwent passive static stretching of the gastrocnemius and soleus muscles (15 repetitions, holding the stretch for 20 s). The control group performed foot exercises on their own (seven exercises, 10–20 repetitions of each exercise). The authors of the study did not specify how often the therapeutic sessions were performed, but they showed that myofascial release combined with foot exercises significantly improved the range of dorsiflexion and plantar flexion as well as inversion and eversion in the ankle joint.

Our study focuses on the problem of flat feet in adults who do not train or engage in any recreational physical activity. To check the effectiveness of myofascial release, this method was compared with foot exercises and a group without intervention. Myofascial release was performed by the therapist’s hand, and the intervention lasted daily for four weeks. The effects of the therapy were assessed using a goniometric test, and both dorsiflexion and plantar flexion of the foot as well as inversion and eversion movements were measured. Our study showed that both myofascial release (MR) alone and myofascial release combined with exercise (MRE) techniques were most effective in improving both foot flexion movements. Additionally, it was shown that myofascial release combined with exercises (MRE) statistically significantly improved the range of described movements compared to myofascial release (MR) techniques alone and, in the case of plantar flexion in both feet, also to the control group (C). The situation was also similar in the case of the assessed inversion and eversion movements. Only groups MRE and MR achieved statistically significant improvement in the range of both movements in both feet simultaneously. In the intergroup comparisons, statistical significance was obtained only for eversion, and groups MRE and ME significantly improved the range of this movement in the left foot compared to the control group.

Another problem that may accompany flat feet in adults is a reduced overall foot function. There are a small number of reports in the available literature that focus on the effectiveness of myofascial release in improving foot function, and the existing ones do not refer to flat feet. In addition to assessing the range of motion in people with foot valgus after using myofascial release, among others, the above-mentioned Gupta et al. [29] also measured foot performance using the Foot Function Index (FFI). Like the results obtained when assessing the range of motion in the ankle joint, they also achieved a significant improvement in foot function after a four-week intervention including myofascial release performed using the NordBlade 2.0 tool. Similar results were obtained by Akter et al. [23] who also assessed foot performance using the FFI. The combined therapy they used (myofascial release, ice compression, stretching exercises) in people with plantar heel pain improved the overall function of the feet after four weeks (12 sessions). In our study, foot function was assessed using the FAOQ [22] as it was validated, culturally adapted and translated into Polish.

Our study has shown that after the therapy, the performance of the feet significantly improved in groups MRE, MR and E (in each group the FAOQ score was above the minimal clinically important difference—MCID), but none were more effective in this activity, either compared to each other or to the control group, which indicates that each type of intervention is equally effective in improving the assessed indicator.

Any differences between our results and those of other authors may be due to the different numbers of people studied, their level of physical activity, as well as the different types of myofascial techniques or assessment tools used.

The topic of the effectiveness of myofascial therapy in the case of flat feet seems to remain open. The results of our study, as well as the relatively small number of available publications on this topic, indicate the need for further research in this direction.

**Study limitation**—this study is not without limitations. Increasing the duration of therapeutic program would be a good guideline for future studies. In further research, the sample size could also be increased to include more people in each group. A good complement to the research would also be assessment regarding the long-term effects of the applied therapeutic interventions.

## 5. Practical Applications

Our study showed that all the applied physiotherapy techniques had an impact on improving the range of motion in the ankle joint and the functional efficiency of the feet, but myofascial release techniques proved to be the most effective, both when used individually and in combination with exercises. Therefore, it seems reasonable to include myofascial release in therapeutic programs dedicated to adults with flat feet, including athletes reporting foot ailments.

## 6. Conclusions

1. In the people studied, the combination of myofascial techniques and exercises (MRE) was the most effective for improving the tested ranges of motion of the ankle joint.
2. In the study participants, myofascial techniques had a significant impact on the performance of the feet assessed with the FAOQ.

## Figures and Tables

**Table 1:** Anthropometric characteristics of the examined persons.

	Group MRE			Group MR			Group E			Group C			Intergroup Comparison
	$\bar{x}$	Min	Max	$\bar{x}$	Min	Max	$\bar{x}$	Min	Max	$\bar{x}$	Min	Max	*p*
Age (yrs)	29.8	20.0	46.0	35.3	25.0	47.0	30.8	22.0	49.0	34.1	20.0	49.0	0.189
Body weight (kg)	70.2	65.0	49.0	69.5	70.0	57.0	71.3	70.0	50.0	69.9	70.0	55.0	0.981
Body height (cm)	170.3	160.0	185.0	168.6	157.0	180.0	170.7	158.0	190.0	171.3	161.0	179.0	0.770
BMI (kg/m^2^)	24.1	18.3	32.7	24.6	20.5	28.7	24.2	17.7	32.2	23.8	20.2	29.7	0.978

$\bar{x}$—arithmetic mean, *p*—level of significance of differences.

**Table 2:** Measurement results of the range of motion of dorsiflexion and plantar flexion of both feet in the examined persons.

Foot	Motion	Group		Before Therapy		After Therapy		Intergroup Comparison (After Therapy)
				$\bar{x}$	SD	$\bar{x}$	SD	*p*, *d*, *m*
Left	Dorsiflexion	MRE		19.20	2.81	21.73	2.52	MRE vs. MR = 0.006 *, 1.074, 0.889 MRE vs. E = 0.271, 0.411, 0.292 MRE vs. C = 0.456, 0.276, 0.182 MR vs. E = 0.054, 0.736, 0.626 MR vs. C = 0.003, 1.207, 0.943 E vs. C = 0.096, 0.629, 0.514
		MR		21.33	3.72	25.27	3.92	
		E		21.40	3.38	22.80	2.68	
		C		20.87	3.62	20.93	3.24	
	Comparison between measurements (*p*, *d*, *m*)			MRE = 0.017 *, 1.481, 0.979; MR = 0.001 *, 1.626, 0.999; E = 0.048 *, 0.690, 0.815; C = 0.872, 0.027, 0.061				
	Plantar flexion	MRE		40.93	3.41	44.07	3.53	MRE vs. MR = 0.045 *, 0.766, 0.656 MRE vs. E = 0. 0001 *, 1.693, 0.998 MRE vs. C = 0.004 *, 1.143, 0.920 MR vs. E = 0.035 *, 0.677, 0.565 MR vs. C = 0.682, 0.153, 0.108 E vs. C = 0.064, 0.702, 0.591
		MR		37.73	4.68	41.07	4.27	
		E		37.73	3.26	38.60	2.90	
		C		39.73	2.76	40.53	2.59	
	Comparison between measurements (*p*, *d*, *m*)			MRE = 0.006 *, 1.429, 0.999; MR = 0.001 *, 1.169, 0.996; E = 0.117, 0.440, 0.491; C = 0.063, 0.471, 0.536				
Right	Dorsiflexion		MRE	19.20	3.34	22.13	2.92	MRE vs. MR = 0.028 *, 0.843, 0.728 MRE vs. E = 1.000, 0.000, 0.05 MRE vs. C = 0.711, 0.137, 0.101 MR vs. E = 0.025 *, 0.863, 0.746 MR vs. C = 0.013 *, 0.970, 0.828 E vs. C = 0.704, 0.140, 0.102
			MR	21.20	2.96	24.73	3.24	
			E	20.87	3.46	22.13	2.77	
			C	21.00	2.70	21.73	2.94	
	Comparison between measurements (*p*, *d*, *m*)			MRE = 0.008 *, 1.451, 0.999; MR = 0.001 *, 1.784, 0.999; E = 0.063, 0.607, 0.722; C = 0.077, 0.406, 0.441				
	Plantar flexion		MRE	38.47	10.49	44.20	3.90	MRE vs. MR = 0.042 *, 0.617, 0.502 MRE vs. E = 0.003 *, 1.175, 0.932 MRE vs. C = 0.027 *, 0.893, 0.771 MR vs. E = 0.683, 0.248, 0.163 MR vs. C = 0.796, 0.176, 0.120 E vs. C = 0.119, 0.049, 0.065
			MR	38.33	5.89	41.20	5.66	
			E	38.00	4.33	40.07	3.08	
			C	39.87	4.76	40.27	4.85	
	Comparison between measurements (*p*, *d*, *m*)			MRE = 0.003 *, 0.741, 0.860; MR = 0.002 *, 0.784, 0.893; E = 0.003 *, 0.788, 0.895; C = 0.043 *, 0.132, 0.123				

$\bar{x}$—arithmetic mean, SD—standard deviation, *p*—level of significance of differences, *—statistically significant differences, *d*—dCohen, *m*—power of test.

**Table 3:** Measurement results of the range of inversion and eversion of both feet in the examined persons.

Foot	Motion	Group	Before Therapy		After Therapy		Intergroup Comparison (After Therapy)
			$\bar{x}$	SD	$\bar{x}$	SD	*p*, *d*, *m*
Left	Inversion	MRE	34.53	5.10	38.47	5.42	MRE vs. MR = 0.130, 0.569, 0.451 MRE vs. E = 0.135, 0.563, 0.444 MRE vs. C = 0.379, 0.326, 0.220 MR vs. E = 0.925, 0.034, 0.060 MR vs. C = 0.356, 0.343, 0.233 E vs. C = 0.379, 0.329, 0.222
		MR	38.27	4.43	41.20	4.09	
		E	39.53	5.13	41.07	3.65	
		C	39.53	3.83	39.93	3.28	
	Comparison between measurements (*p*, *d*, *m*)		MRE = 0.003 *, 1.179, 0.996; MR = 0.001 *, 1.080, 0.990; E = 0.074, 0.495, 0.571; C = 0.334, 0.173, 0.157				
	Eversion	MRE	28.60	3.60	32.13	2.75	MRE vs. MR = 0.690, 0.148, 0.106 MRE vs. E = 0.110, 0.602, 0.486 MRE vs. C = 0.004 *, 1.150, 0.923 MR vs. E = 0.079, 0.668, 0.556 MR vs. C = 0.005 *, 1.113, 0.908 E vs. C = 0.195, 0.482, 0.361
		MR	29.87	4.12	32.60	3.56	
		E	29.47	2.77	30.53	2.56	
		C	28.53	2.53	29.47	1.77	
	Comparison between measurements (*p*, *d*, *m*)		MRE = 0.001 *, 1.163, 0.996; MR = 0.001 *, 1.098, 0.992; E = 0.012, 0.625, 0.744; C = 0.069, 0.611, 0.727				
Right	Inversion	MRE	35.53	3.54	38.87	4.21	MRE vs. MR = 0.336, 0.356, 0.244 MRE vs. E = 0.639, 0.172, 0.118 MRE vs. C = 0.699, 0.142, 0.103 MR vs. E = 0. 630, 0.177, 0.121 MR vs. C = 0.486, 0.257, 0.169 E vs. C = 0.885, 0.053, 0.066
		MR	36.00	4.60	40.33	3.99	
		E	38.40	5.00	39.60	4.26	
		C	39.40	3.25	39.40	3.20	
	Comparison between measurements (*p*, *d*, *m*)		MRE = 0.008 *, 1.319, 0.999; MR = 0.001 *, 1.559, 0.999; E = 0.025 *, 0.399, 0.431; C = 1.000, 0.000, 0.050				
	Eversion	MRE	27.40	3.68	30.00	4.38	MRE vs. MR = 0.026 *, 0.856, 0.740 MRE vs. E = 0.114, 0.596, 0.479 MRE vs. C = 0.115, 0.592, 0.475 MR vs. E = 0.359, 0.339, 0.230 MR vs. C = 0.146, 0.547, 0.427 E vs. C = 0.710, 0.140, 0.102
		MR	32.27	4.35	33.40	3.52	
		E	31.00	3.32	32.27	3.13	
		C	31.73	1.87	31.93	1.44	
	Comparison between measurements (*p*, *d*, *m*)		MRE = 0.004 *, 0.987, 0.976; MR = 0.029 *, 0.433, 0.480; E = 0.009 *, 0.620, 0.738; C = 0.629, 0.178, 0.161				

$\bar{x}$—arithmetic mean, SD—standard deviation, *p*—level of significance of differences, *—statistically significant differences, *d*—dCohen, *m*—power of test.

**Table 4:** Results of the FAOQ in the surveyed people.

	Group	Before Therapy		After Therapy		Intergroup Comparison (After Therapy)
		$\bar{x}$	SD	$\bar{x}$	SD	*p*, *d*, *m*
FAOQ	MRE	80.73	10.92	87.53	8.98	MRE vs. MR = 0.357, 0.342, 0.232 MRE vs. E = 0.569, 0.210, 0.139 MRE vs. C = 0.944, 0.025, 0.057 MR vs. E = 0.866, 0.062, 0.070 MR vs. C = 0.403, 0.309, 0.206 E vs. C = 0.571, 0.209, 0.139
	MR	78.47	10.60	90.13	5.93	
	E	83.53	8.66	89.60	10.63	
	C	83.20	12.16	87.27	11.68	
Comparison between measurements (*p*, *d*, *m*)		MRE = 0.010 *, 1.037, 0.985; MR = 0.001 *, 1.702, 0.999; E = 0.015 *, 0.951, 0.968; C = 0.116, 0.539, 0.633				

$\bar{x}$—arithmetic mean, SD—standard deviation, *p*—level of significance of differences, *—statistically significant differences, *d*—dCohen, *m*—power of test.

## Data Availability

Data used in this paper are available from the principal investigator.
